# Common Arterial Trunk with Intact Ventricular Septum: Morphologic and Developmental Considerations

**DOI:** 10.3390/jcdd13070288

**Published:** 2026-06-23

**Authors:** Rohit S. Loomba, Diane E. Spicer, Robert H. Anderson

**Affiliations:** 1 Ann & Robert H. Lurie Children’s Hospital, Northwestern University Feinberg School of Medicine, Chicago, IL 60611, USA; 2 Johns Hopkins All Children’s Hospital, St. Petersburg, FL 33701, USA; dspicer6@jhmi.edu; 3 Biosciences Institute, Newcastle University, Newcastle-upon-Tyne NE2 4HH, UK; sejjran@ucl.ac.uk

**Keywords:** common arterial trunk, truncus arteriosus, intact ventricular septum, ventricular septal defect, congenital heart disease

## Abstract

Background: It is rare in clinical practice to encounter a common arterial trunk when the ventricular septum is intact. In this setting, other clinical diagnoses, such as hypoplastic left heart syndrome with aortic atresia, may be mistaken for a common arterial trunk. Data for this combination is largely limited to case reports and small case series. We have conducted a systematic review of reported cases, performing cluster analyses to provide an objective grouping of the cases. Methods: A systematic review of the literature was performed to identify cases of a common arterial trunk with an intact ventricular septum. Cases for which individual data were available were included in the final analyses. Cluster analysis using K-means clustering was conducted to provide an objective grouping of the hearts based on morphologic findings. Results: K-means clustering identified three distinct groups among hearts with a common arterial trunk with intact ventricular septum. The commitment of the common ventriculo-arterial junction to the left, right, or both ventricles was the defining feature of each group. Hearts with a common trunk committed to one of the ventricles demonstrated significant hypoplasia or atresia of structures related to the other ventricle. Conclusions: Distinct patterns can be identified when a common arterial trunk is found with an intact ventricular septum. They depend on the ventricle or ventricles, which support the common ventriculo-arterial junction.

## 1. Introduction

Common arterial trunk with an intact ventricular septum is a rare clinical entity in which the solitary arterial trunk that gives rise to the coronary arteries, the pulmonary arteries, and the intrapericardial aorta arises from either the left ventricle, the right ventricle, or both ventricles, while the ventricular septum is intact. In most instances, a common arterial trunk is associated with a juxta-arterial outlet interventricular communication. The presence of such a defect, however, is not an integral part of the phenotypic feature of a common arterial trunk, which is the commonality of the ventriculo-arterial junction. In this setting, some examples can be found with an intact ventricular septum. In such hearts, there is mixing and shunting of the ventricular outlets within the common arterial trunk at the arterial level before the blood enters either the systemic or pulmonary circulations. In the examples in which the common ventriculo-arterial junction remains supported by both ventricles, a fibrous partition joining two of the leaflets of the common arterial valve is attached to the crest of the muscular ventricular septum, thus dividing the common valve into discrete parts for the outflow tracts of the right and left ventricles. In this setting, the arrangement of the common ventriculo-arterial junction can be compared to the common atrioventricular junction, as found in atrioventricular septal defect with exclusive atrial or ventricular shunting, in the sense that, in both situations, there is a common junction guarded by a common valve, whereas usually there are two junctions and two valves. As we will describe, however, the common trunk can also be found with an intact ventricular septum when the common junction is supported exclusively by either the right or the left ventricle. Our aim was to review all reported cases of a common arterial trunk with an intact ventricular septum to establish morphologically based clusters.

## 2. Materials and Methods

### 2.1. Study Design

We performed a systematic review of the published literature to identify and characterize reported cases of common arterial trunk with an intact ventricular septum [1,2,3,4,5,6,7,8,9,10,11,12,13,14]. As our exercise was a systematic review using already published de-identified data from other institutions, institutional review board approval was not required.

### 2.2. Systematic Review

PubMed, OVID, and Medline were queried using the following keywords for the literature review: “common arterial trunk”, “truncus arteriosus”, “truncus”, “intact”, and “ventricular septum”. These terms were used in isolation and in various combinations across all databases. The resulting studies were then screened by title and abstract to determine the relevance of the identified publications. Those deemed relevant after this screen then had their full text reviewed. Studies were included in the final analyses if they properly accounted for hearts with a common arterial trunk and an intact ventricular septum and included data relating to the individual hearts, as opposed to simple summative data.

### 2.3. Data Collection

Patient data, including sex and age at diagnosis, was collected. Morphologic data included all components of a sequential segmental analysis, including all aspects of cardiac anatomy. Clinical data included whether surgical repair was undertaken, the last age at follow-up, the presence of a genetic anomaly, and whether or not the patient was alive at the last follow-up.

### 2.4. Statistical Analyses

Descriptive statistics were tabulated for the cohort. Frequencies and percentages were calculated for descriptive characteristics, while the mean and standard deviation were calculated for continuous variables. A cluster analysis was conducted including only morphologic and clinical data. This was done utilizing a K-means clustering approach. An elbow method was used to determine the optimal number of clusters by plotting the within-cluster sum of squares against the number of clusters, identifying the point where the rate of decrease sharply changes.

## 3. Results

### 3.1. Cohort Characteristics 

We found details of 14 hearts satisfying the criteria for inclusion in our final analyses. Of these, 8 (57%) were from male patients (Table 1). Of the overall series, 9 patients had undergone surgical palliation or repair. Of these 9 surgically repaired hearts, a biventricular repair had proved possible in five. A genetic anomaly, specifically 22q deletion, was reported in 2 (14%) of the overall cases. The specific genetic testing done is not known so the absence of a reported genetic anomaly does not necessarily indicate the absence of such an anomaly.

### 3.2. Cardiac Morphology

The systemic and pulmonary venous returns were usual in all the hearts, as were the atrial arrangement and atrioventricular connections. Deficient atrial septation was reported in 12 (86%) instances, all of them representing a defect within the oval fossa. The tricuspid valve was reported as abnormal in three (21%) hearts, being displaced, hypoplastic, and atretic in one heart each. The mitral valve was described as abnormal in six (43%) hearts, being hypoplastic in three hearts and atretic in three hearts. The ventricular mass in all hearts exhibited right-handed topology, with the ventricular apex pointing leftward. The right ventricle was hypoplastic in three (21%) of the hearts, while the left ventricle was hypoplastic in four. In six of the hearts, the common ventriculo-arterial junction was shared between the ventricles (43%), but arose exclusively from the right ventricle in five (36%), and from the left ventricle in three (21%). In half of the hearts, the truncal valve was trifoliate, but was quadrifoliate in four (29%), biofoliate in two (14%), and described as having six leaflets in one (7%). No details were provided, however, regarding the number of valvar sinuses. The aortic arch was left-sided in 11 (79%) and right-sided in three (21%). Interruption of the aortic arch was present in two (14%) hearts, with these hearts having pulmonary as opposed to aortic dominance. The origins of the head and neck vessels were abnormal in four (29%) hearts. Flow to the lungs was through right and left pulmonary arteries with separate orifices from the common trunk in 10 (71%) of the hearts, with a confluent pulmonary segment described in the remaining four. An arterial duct was described in both of the hearts with interruption of the aortic arch. In the setting of a common arterial trunk, there is no “usual” arrangement for the coronary arteries. Amongst the descriptions, however, we found one account of a solitary coronary artery, and another of right coronary arterial ostial atresia with ventriculo-coronary fistulous connections.

### 3.3. Cluster Analyses

**Figure 1:**
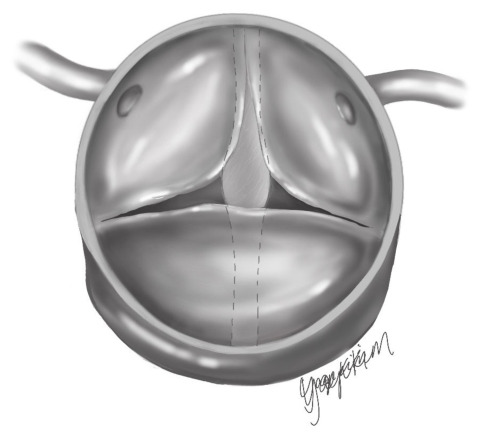
An illustration demonstrating the ventriculo-arterial junction in the short axis. The junction is being viewed from the superior aspect downwards. The ventricular septum is depicted below the truncal valve. Illustration by Yaeji Kim.

**Figure 2:**
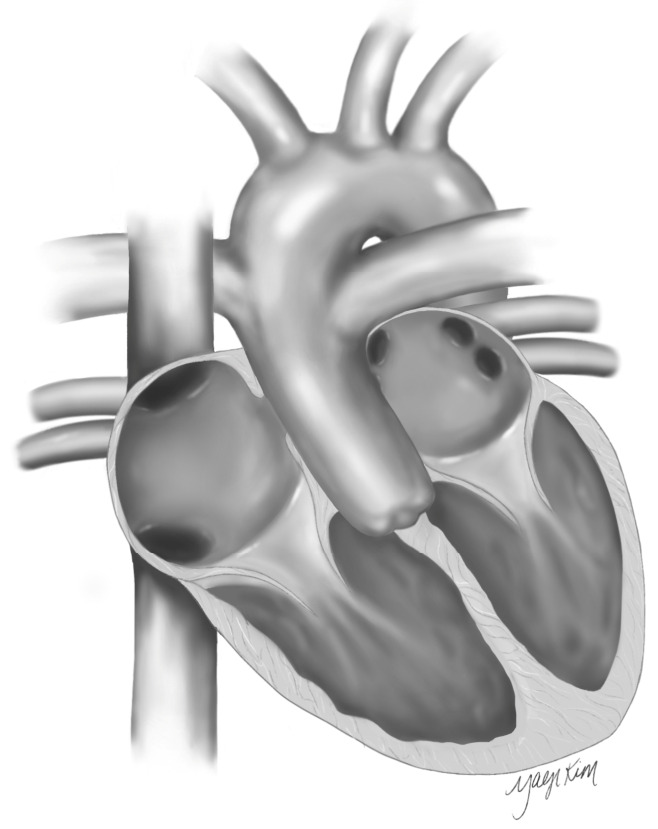
An illustration demonstrating the characteristic figures of hearts in cluster 1.

Cluster analysis revealed three clusters, which depended on the support provided to the ventriculo-arterial junction (Figure 1). The first cluster included hearts in which the ventriculo-arterial junction was supported by both ventricles. All of these hearts had normal atrioventricular valves, with the ventricles being of normal size. Coronary arterial anomalies, and one of the instances of interruption of the aortic arch, were included in this cluster. A majority of hearts in this cluster had right and left pulmonary arteries which arose from separate orifices from the arterial trunk. A biventricular repair was deemed possible in a majority of cases making up this cluster (Figure 2).

**Figure 3:**
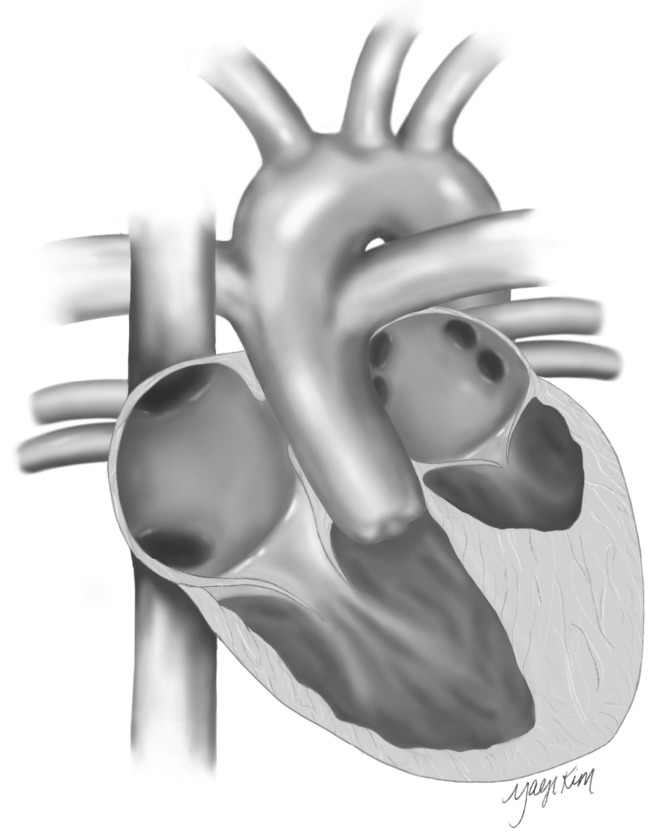
An illustration demonstrating the characteristic figures of hearts in cluster 2.

The second cluster was made up of hearts with the common ventriculo-arterial junction supported by the right ventricle. All of these hearts had co-existing mitral atresia and a hypoplastic left ventricle, albeit with normal tricuspid valves and right ventricles. The other heart with interruption of the aortic arch was included in this cluster. A majority of hearts had right and left pulmonary arteries arising through separate orifices from the common trunk. All of these patients were female, and none underwent a biventricular repair. The two patients with genetic anomalies were included in this cluster (Figure 3).

**Figure 4:**
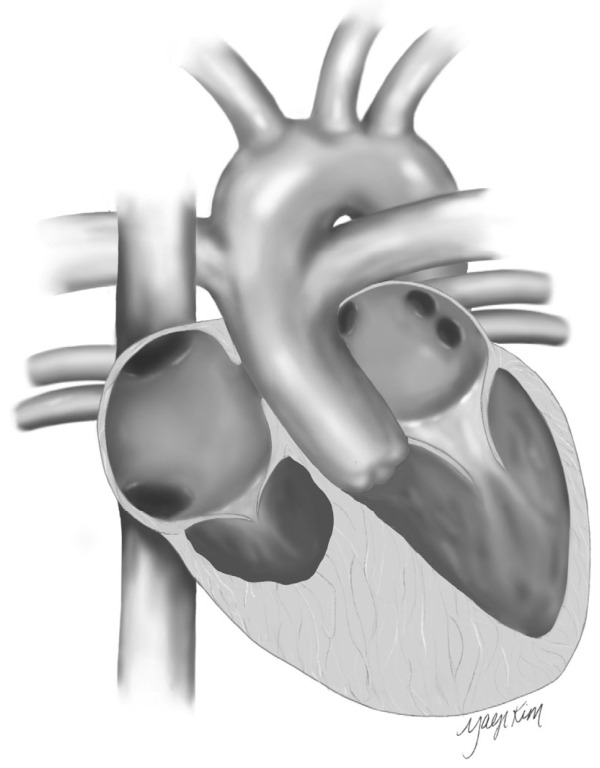
An illustration demonstrating the characteristic figures of hearts in cluster 3.

The third cluster was made up of hearts with the ventriculo-arterial junction supported exclusively by the left ventricle. The mitral valve was described as being hypoplastic in two-thirds of these hearts, although the left ventricle was of normal size in all. The tricuspid valve was abnormal in all, with the right ventricle often described as hypoplastic. The aortic arch was right-sided in two-thirds of this cluster, but none of the hearts had aortic interruption. As with those having exclusively right ventricular origin of the trunk, a biventricular repair had not been attempted in any of these patients (Figure 4).

The other important feature identified by cluster analysis was whether the mitral valve was normal or abnormal, and whether the left ventricle was hypoplastic. Figure 2, Figure 3 and Figure 4 illustrate the usual findings across the three clusters.

## 4. Discussion

We have collated findings from previous descriptions of the rare combination of common arterial trunk with an intact ventricular septum, [1,2,3,4,5,6,7,8,9,10,11,12,13,14] undertaking a novel cluster analysis to help better understand the major phenotypes. The feature identified by the analysis as distinguishing between the groups is the ventricular origin of the common ventriculo-arterial junction. Those hearts with the common junction arising from both ventricles tended to have normal tricuspid and mitral valves, along with normally sized ventricles. Hearts with the common junction supported exclusively by the right ventricle tended to have mitral atresia and hypoplastic left ventricles. Those with the common junction arising exclusively from the left ventricle tended to have abnormal tricuspid valves, a hypoplastic right ventricle, and a right-sided aortic arch. In all the hearts, nonetheless, the pulmonary blood flow was usually from right and left pulmonary arteries that arose by separate orifices from the common trunk. The feature of all the hearts is the shunting of blood at an arterial level. Irrespective of the ventricular origin of the trunk, the blood that enters the trunk can shunt or mix prior to entering the systemic or pulmonary circulation. Thus, even though, because of the integrity of the ventricular septum, there is no shunting at the ventricular level, a shunt remains at the arterial level, this being an important clinical finding.

In those hearts in which the common junction is supported by both ventricles, the characteristic feature is the presence of a fibrous shelf extending from the leaflets of the valve and attached to the crest of the scooped-out muscular ventricular septum. In this way, the shelf divides the orifice of the common trunk into separate right and left components, with each component committed to its own ventricle. It is this arrangement that provides an explanation as to how, in one of the hearts, the truncal valve was described as possessing six leaflets, with this arrangement produced by the shelf uniting two of the leaflets of an initially quadrifoliate valve. The overall arrangement can be compared to the situation in the so-called “ostium primum” defect. In this latter setting, a tongue of fibrous tissue joins the bridging leaflets of a common atrioventricular valve in the presence of an atrioventricular septal defect, thus producing separate atrioventricular valves within a common atrioventricular junction.

To appreciate how a comparable arrangement can be found when there is a common arterial trunk, it is necessary to understand the development of the outflow tracts. Historically, the development of the outflow tracts was described using a framework consisting of the “conus” and “truncus”, although there have never been discrete and widely accepted definitions of these terms. The mechanism of the separation of the initially solitary outflow tract into these components was arbitrary, without much objective developmental data underpinning the basic notion. A “conotruncal” framework, furthermore, ignores the development of the arterial roots and valves, which are unequivocally part of the definitive outflow tracts. An episcopic study of mouse embryos has now allowed for objective observation of the pertinent development [15]. This shows that development is best described in a tripartite fashion, recognizing proximal, middle, and distal segments of the outflow tracts. It is the migration of neural crest cells, along with the ingrowth of non-myocardial components, that underscores the developmental processes. The growth of non-myocardial tissue into the pericardial cavity eventually leads to the distal separation of the intrapericardial arterial trunks. As part of this process, an aortopulmonary septum, packed with neural crest cells, grows into the pericardial cavity from the dorsal wall of the aortic sac, ultimately fusing with the distal outflow cushions, themselves also packed with cells derived from the neural crest. The end result is the elimination of the embryonic aortopulmonary foramen. Once this embryonic foramen is closed, the outflow tract is separated into its aortic and pulmonary components. Deficiencies of this septation can self-evidently produce an aortopulmonary window but can also result in the persistence of a common arterial trunk [15].

While the aortopulmonary foramen is closing, the distal cushions are themselves fusing in what can then be recognized as the middle part of the outflow tract. At a similar point in development, there has also been formation of the intercalated valvar swellings. The distal ends of the cushions and the intercalated swellings ultimately excavate to form the aortic and pulmonary valvar leaflets, with the adjacent leaflets arising from the cushions, and the non-adjacent leaflets from the intercalated swellings. If the distal cushions do not fuse, there will be a common arterial valve. It is then the extent of hypoplasia of the intercalated swellings that determines the variability in the number of leaflets making up the common valve. It is noteworthy that Anderson and colleagues had demonstrated failure of fusion of the outflow cushions as the reason for the persistence of a common arterial trunk using dogs several decades ago [15]. In that the distal cushions excavate to produce valvar leaflets, the proximal parts of the cushions, if development proceeds normally, fuse with each other and with the crest of the ventricular septum so as to commit the aortic root to the left ventricle. The shelf thus formed muscularises to become the free-standing subpulmonary infundibulum. Should the cushions fuse but fail to muscularise, the end result is a juxta-arterial ventricular septal defect, which also has as part of its phenotypic make-up a common ventriculo-arterial junction [15]. The juxta-arterial defect, therefore, is also part of the family of lesions sharing a potential ventriculo-arterial septal defect [1]. In the majority of cases having the ventriculo-arterial defect, the valve guarding the co-existing common ventriculo-arterial junction is itself common. This arrangement, of course, is found in the majority of patients having a common arterial trunk, in which there is the potential for shunting at both ventricular and arterial levels. In the setting of the juxta-arterial ventricular septal defect, the shunting is exclusively at the ventricular level. It is in the rare examples of a common arterial trunk with an intact ventricular septum in which the shunting is exclusively at the arterial level. These examples, however, are not aortopulmonary windows, since although there can be separate valvar components for the right and left ventricles, the valvar parts are housed within a common arterial root. The phenotypic feature of aortopulmonary windows is the presence of separate aortic and pulmonary roots. We should also emphasize that, while the hearts included in our current analyses have unequivocal left ventricular hypoplasia, and also have an intact ventricular septum, they are not examples of hypoplastic left heart syndrome. The aorta in all our examples arises from the common arterial trunk, rather than from the hypoplastic left ventricle.

It was by use of cluster analyses that we were able objectively to classify these rare combinations. Such an approach is arguably the ideal system for classification. Arbitrary schemes are less likely to be inclusive or efficient. A good example of an arbitrary classification is the scheme offered by Collett and Edwards for “truncus arteriosus”. The classification did not take into account the presence or absence of a ventricular septal defect, although in its defense, the hearts with an intact septum had not been described at the time. The scheme did include, nonetheless, hearts that now are better classified as part of the grouping of tetralogy with pulmonary atresia. Proper description of congenitally malformed hearts is now essential if we are to share accurate and complete clinical information. If classification is deemed necessary, then it, in turn, should be based on an objective evaluation of the data. We have shown that such an objective classification is possible even when considering a common arterial trunk in the setting of an intact ventricular septum, which is a remarkably rare anatomic combination.

## Figures and Tables

**Table 1:** Outlines the cohort characteristics.

Variable	Cluster 1 (*n* = 6)	Cluster 2 (*n* = 5)	Cluster 3 (*n* = 3)
Age at diagnosis, months	7.8 (6.4)	3.1 (2.7)	17.5 (16.3)
Age at last follow-up, months	48.6 (22.1)	31.4 (18.6)	12.0 (5.7)
Female	1 (16.7%)	5 (100%)	0 (0%)
Mortality	0 (0%)	1 (20.0%)	1 (33.3%)
Biventricular repair	5 (83.3%)	0 (0%)	0 (0%)
Arterial duct present	1 (16.7%)	1 (20.0%)	0 (0%)
Tricuspid valve abnormal	0 (0%)	0 (0%)	3 (100%)
Mitral valve abnormal	0 (0%)	5 (100%)	1 (33.3%)
Right ventricle hypoplastic	0 (0%)	0 (0%)	3 (100%)
Left ventricle hypoplastic	0 (0%)	5 (100%)	0 (0%)
Ventricular topology: right-handed	6 (100%)	5 (100%)	3 (100%)
Cardiac apex leftward	6 (100%)	5 (100%)	3 (100%)
Aortic arch left-sided	5 (83.3%)	3 (60.0%)	1 (33.3%)
Pulmonary dominance	5 (83.3%)	4 (80.0%)	1 (33.3%)
Aortic dominance	1 (16.7%)	1 (20.0%)	2 (66.7%)
Truncal valve dysplasia/abnormality	1 (16.7%)	3 (60.0%)	2 (66.7%)
Truncal valve regurgitation (moderate or greater)	0 (0%)	2 (40.0%)	1 (33.3%)
Truncal valve stenosis	0 (0%)	1 (20.0%)	1 (33.3%)
Interrupted aortic arch/arch obstruction	1 (16.7%)	1 (20.0%)	0 (0%)
Coronary anomaly present	2 (33.3%)	0 (0%)	0 (0%)

## Data Availability

No new data were created or analyzed in this study.

## References

[1] Spicer D.E., Steffensen T.S. (2020). A Rare Presentation of Common Arterial Trunk with Intact Ventricular Septum. J. Cardiovasc. Dev. Dis..

[2] Ajami G., Amirghofran A.A., Amoozgar H., Borzouee M. (2015). Persistent Truncus Arteriosus with Intact Ventricular Septum: Clinical, Hemodynamic and Short-term Surgical Outcome. Iran. J. Pediatr..

[3] Alves P.M., Ferrari A.H. (1987). Common arterial trunk arising exclusively from the right ventricle with hypoplastic left ventricle and intact ventricular septum. Int. J. Cardiol..

[4] Carr I., Bharati S., Kusnoor V.S., Lev M. (1979). Truncus arteriosus communis with intact ventricular septum. Br. Heart J..

[5] Chikkagoudar K., Gupta P., Koneti N., Dash T., Doraiswamy V. (2020). Common arterial trunk with intact septum and hypoplastic right ventricle: An uncommon embryological entity. Ann. Pediatr. Cardiol..

[6] Davidson H., Seco M., Asakai H., Liava’a M. (2023). A case report of truncus arteriosus with intact ventricular septum and crossed branch pulmonary arteries. Eur. Heart J.-Case Rep..

[7] Garg P., Mishra A., Shah R., Parmar D., Anderson R.H. (2015). Neonatal Repair of Common Arterial Trunk with Intact Ventricular Septum. World J. Pediatr. Congenit. Heart Surg..

[8] Loeffelbein F., Wronski L., Riede F.T. (2023). Echocardiography: Conotruncal anomaly: A case of common arterial trunk with intact ventricular septum and hypoplastic left heart complex with unbalanced pulmonary stenoses. Cardiol. Young.

[9] Marathe S.P., Naganur S.H., Menon S., Orr Y., Cooper S.G., Winlaw D.S. (2018). An Unusual Combination of Truncus Arteriosus, Interrupted Aortic Arch, and Hypoplastic Left Ventricle. World J. Pediatr. Congenit. Heart Surg..

[10] McElhinney D.B., Reddy V.M., Brook M.M., Hanley F.L. (1997). Repair of truncus arteriosus with intact ventricular septum (Van Praagh type B2) in a neonate. J. Thorac. Cardiovasc. Surg..

[11] Michelfelder E.C., Zales V.R., Jacobs M.L. (1998). Surgical palliation of truncus arteriosus with mitral atresia and hypoplastic left ventricle. Ann. Thorac. Surg..

[12] Sandoval Boburg R., Hahn J.K., Hofbeck M., Schlensak C. (2020). Persistent common arterial trunk with hexaleaflet truncal valve and intact ventricular septum. Interact. Cardiovasc. Thorac. Surg..

[13] Zeevi B., Dembo L., Berant M. (1992). Rare variant of truncus arteriosus with intact ventricular septum and hypoplastic right ventricle. Br. Heart J..

[14] Zhang Y.Q., Shen R., Sun K., Zhong S.W., Wu Y.R. (2009). Persistent truncus arteriosus with intact ventricular septum diagnosed by echocardiography. Chin. Med. J..

[15] Anderson R.H., Lamers W.H., Hikspoors J.P.J.M., Mohun T.J., Bamforth S.D., Chaudhry B., Eley L., Kerwin J., Crosier M., Henderson D.J. (2024). Development of the arterial roots and ventricular outflow tracts. J. Anat..

